# Acetylation-dependent regulation of essential iPS-inducing factors: a regulatory crossroad for pluripotency and tumorigenesis

**DOI:** 10.1002/cam4.298

**Published:** 2014-08-13

**Authors:** Xiangpeng Dai, Pengda Liu, Alan W Lau, Yueyong Liu, Hiroyuki Inuzuka

**Affiliations:** Department of Pathology, Beth Israel Deaconess Medical Center, Harvard Medical SchoolBoston, Massachusetts

**Keywords:** Akt, iPS cell, Klf4, Oct4, p300, Sox2

## Abstract

Induced pluripotent stem (iPS) cells can be generated from somatic cells by coexpression of four transcription factors: Sox2, Oct4, Klf4, and c-Myc. However, the low efficiency in generating iPS cells and the tendency of tumorigenesis hinder the therapeutic applications for iPS cells in treatment of human diseases. To this end, it remains largely unknown how the iPS process is subjected to regulation by upstream signaling pathway(s). Here, we report that Akt regulates the iPS process by modulating posttranslational modifications of these iPS factors in both direct and indirect manners. Specifically, Akt directly phosphorylates Oct4 to modulate the Oct4/Sox2 heterodimer formation. Furthermore, Akt either facilitates the p300-mediated acetylation of Oct4, Sox2, and Klf4, or stabilizes Klf4 by inactivating GSK3, thus indirectly modulating stemness. As tumorigenesis shares possible common features and mechanisms with iPS, our study suggests that Akt inhibition might serve as a cancer therapeutic approach to target cancer stem cells.

## Introduction

Embryonic stem (ES) cells are derived from the inner cell mass of mammalian blastocysts and have been demonstrated to maintain pluripotency 1,2. The pluripotent potential of human ES cells has been proposed to be beneficial in the treatment of various human diseases including Parkinson's disease, spinal cord injury, and diabetes 3,4. However, the therapeutic use of ES cells requires a comprehensive understanding of the molecular mechanisms that control the proliferation and differentiation of ES cells 5,6. More importantly, ethical controversy regarding the use of human embryos also prevents its therapeutic application. To overcome this issue, coexpression of Oct4, Sox2, Klf4, and Nanog or c-Myc in normal mouse or human fibroblasts has been shown to actively reprogram these differentiated cells into an induced pluripotent stem (iPS) cell state 2,7–9. It has been further demonstrated that pluripotency can be induced without c-Myc, however, with lower efficiency 10,11. This provides a manageable approach to generate large quantities of human iPS cells for therapeutic purposes 12. However, the low efficiency of this iPS-generating process hinders the development of iPS technology into clinical applications. Although multiple methods have been attempted to enhance the iPS process, such as adding SV40 large T antigen 13, co-overexpressing MyoD 14, enhancing RA signaling 15, or depleting Mdb3 that is a core component of the NuRD complex 16, it remains largely unknown how the iPS process is regulated by upstream signaling pathways in vivo 17.

Another critical issue that limits the potential application of iPS technology is that all four transcription factors (Sox2, Oct4, Klf4, and c-Myc) are found to be frequently overexpressed in various cancers 6. Thus, it is not surprising that a high percentage of mice derived from iPS cells developed tumors 2. Moreover, it is noteworthy that the iPS process, which requires the coexpression of four transcriptional factors, mirrors the transformation of primary human cells, in which overexpression of the Ha-Ras and hTERT oncogenes and inactivation of both the p53 and Rb tumor suppressor pathways by SV40 LT antigen are required 18,19. More interestingly, tumorigenesis and embryonic development share many similarities. For example, both of them are immortalized and could form tumors when implanted subcutaneously in mice. There is also a growing body of evidence suggesting that many tumors arise by acquiring genetic mutations in only a small population of transformed cells termed cancer stem cells, or cancer initiating cells 20–22. As cancer stem cells are more resistant to common chemotherapeutic interventions, it is critical to understand their biological features in order to develop better anticancer regimens 23,24. To this end, it has been proposed that cancer stem cells were derived from either normal stem cells through acquiring genetic mutations or terminally differentiated somatic cells by activating a subset of genes typically overexpressed in stem cells to acquire a stem cell-like phenotype 24. In this scenario, cellular transformation correlates with the dedifferentiation process. Nonetheless, in both cases genetic mutations are a driving force to cancer stem cell formation. Thus, acquiring mutations that promote tumorigenesis might also partially convert somatic cells into a stem cell-like phenotype through pathways partially resembling the iPS process. In support of this idea, recently it has been shown that inactivation of the p53 tumor suppressor pathway greatly enhanced iPS efficiency 25–29, suggesting that key signaling pathway(s), frequently altered in human cancers, might also be involved in stem cell maintenance 30.

In addition to p53, the PTEN/PI3K/Akt pathway is found commonly hyperactivated in various human carcinomas through various means of genetic alterations and is considered as a hallmark of cancer 31,32. In support of a critical role for Akt in stem cell regulation, constitutive activation of Akt was shown to be capable of substituting for basic fibroblast growth factor 33 or leukemia inhibitory factor to maintain stemness. Consistently, loss of *PTEN* is found to affect the hematopoietic stem cell renewal process 34,35. However, further in-depth evaluations revealed that PTEN negatively regulated the mTORC2/Akt signaling only in adult, but not neonatal hematopoietic stem cells 34. This not only highlighted a development stage-dependent role for PTEN in maintaining stemness but also suggested a potential temporal regulation difference between stem cell self-renewal and tumorigenesis. However, even though Akt has been characterized as a driving oncogene to facilitate tumorigenesis, it remains largely elusive how Akt participates in stem cell fate regulation and whether similar to its oncogenic role, Akt could enhance the efficiency of the iPS process.

## Methods

### Plasmids

CMV-Flag-Sox2, CMV-Flag-Oct4, CMV-Flag-Klf4, and CMV-Flag-Nanog were obtained from Addgene (Cambridge, MA). pcDNA3-HA-p300, Myc-p300, and pcDNA3-HA-CBP were obtained from Dr. James DeCaprio (Dana-Farber Cancer Institute, Boston, MA). pcDNA3-HA-Myr-Akt1 construct was obtained from Dr. Alex Toker (Beth Israel Deaconess Medical Center, Boston, MA) and described previously 36. ERK1, p38-mitogen-activated protein kinase (MAPK), GSK3, and HA-Fbw7 expression plasmids were described previously 37. Various mutation constructs of Flag-Klf4, Flag-Oct4, and Flag-Sox2 were generated using the QuikChange XL Site-Directed Mutagenesis Kit (Stratagene, La Jolla, CA) according to the manufacturer's instructions.

### siRNAs

Scramble, luciferase, *β*-TRCP1+2, Fbw7, Skp2, and Cdh1 siRNA oligos and siRNA transfection methods have been described previously 36.

### Antibodies

Anti-*β*-catenin, Skp2, cyclin E, and polyclonal anti-HA antibodies were purchased from Santa Cruz (Dallas, TX). Anti-phospho-Akt substrate (RxRxxpS/T), acetylated-Lys, and Klf4 antibodies were purchased from Cell Signaling Technology (Danvers, MA). Anti-Tubulin and Vinculin antibodies, polyclonal and monoclonal anti-Flag antibodies, anti-Flag agarose beads, anti-HA agarose beads, and anti-mouse and rabbit horseradish peroxidase-conjugated secondary antibodies were purchased from Sigma (St. Louis, MO). Monoclonal anti-HA antibody was purchased from Covance (Princeton, NJ). Anti-GFP and Cdh1 antibodies were purchased from Invitrogen (Carlsbad, CA).

### Immunoblots and immunoprecipitation

Cells were harvested with EBC buffer (50 mmol/L Tris pH 7.5, 120 mmol/L NaCl, 0.5% NP-40) containing protease inhibitors (Roche, Indianapolis, IN) and phosphatase inhibitors (Calbiochem, Billerica, MA). Whole cell lysates were subjected to immunoblot analyses with indicated antibodies. Immunoprecipitations were carried out by incubating 1 mg of whole cell lysates with 8 *μ*L of HA or Flag slurry beads (Sigma) for 3–4 h at 4°C. Immunoprecipitants were washed five times with NETN buffer (20 mmol/L Tris, pH 8.0, 100 mmol/L NaCl, 1 mmol/L ethylenediaminetetraacetic acid, and 0.5% NP-40) and resolved by sodium dodecyl sulfate polyacrylamide gel electrophoresis (SDS-PAGE) for immunoblot analyses.

### Cell culture and transfection

Cell culture and transfection procedures have been described previously 36,38. For cell transfection, cells were transfected using Lipofectamine (Life Technologies, Woburn, MA) in OptiMEM medium (Life Technologies) according to the manufacturer's instructions. Forty-eight hours posttransfection, transfected cells were further subjected to immunoblot analysis.

## Results

### Oct4 and Klf4 are phosphorylated by Akt in vivo

**Figure 1:**
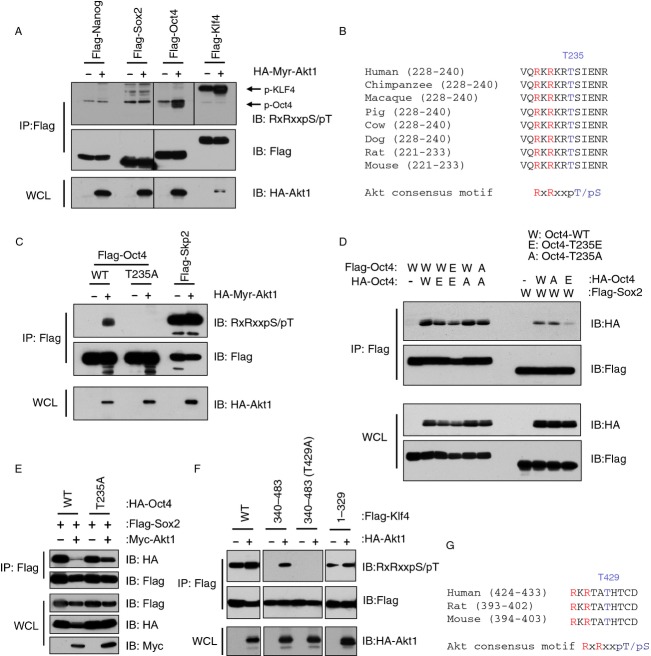
Oct4 and Klf4 are phosphorylated by Akt in vivo. (A) Immunoblot (IB) analysis of whole cell lysates (WCLs) and immunoprecipitates (IPs) derived from 293T cells transfected with HA-tagged Myr-Akt1 and indicated Flag-tagged constructs. Akt-mediated phosphorylation was recognized by an Akt substrate-motif phosphorylation-specific antibody (RxRxxpS/pT). (B) Sequence alignment of the Thr235 putative Akt phosphorylation site in Oct4 among different species. (C) Akt specifically phosphorylated Oct4 at Thr235. (D) Phosphomimetic mutation at Thr235 of Oct4 decreased Oct4 interaction with Sox2. IB analysis and Flag-IP derived from 293T cells transfected with indicated constructs. (E) Phosphorylation of Oct4 on Thr235 led to attenuated Oct4 interaction with Sox2. WCLs of 293T cells transfected with indicated constructs were subjected to immunoprecipitation with Flag antibody. The Flag-IPs and WCLs were immunoblotted with indicated antibodies. (F) Akt phosphorylated Klf4 at Thr399. WCLs of 293T cells transfected with indicated constructs were subjected to immunoprecipitation with Flag antibody. The Flag-IPs and WCLs were immunoblotted with indicated antibodies. (G) Sequence alignment of the Thr429 putative Akt phosphorylation site in Klf4 among different species.

Consistent with previous reports 39,40, we observed that a constitutive active Akt (N-terminal tagged with a myristoylation tag) could phosphorylate Oct4 in cells. Furthermore, Klf4, in addition to Oct4, but not Sox2 or Nanog, was also found phosphorylated by exogenous Akt1 (Fig.1A). By scanning the Oct4 protein sequence, we identified an AGC kinase consensus motif “RxRxxpS/pT” 41 located at the Thr235 residue (Fig.1B) and mutation of this site to a nonphosphorylatable residue, Ala, almost completely abolished Akt1-mediated Oct4 phosphorylation (Fig.1C). The critical function of Oct4 in stem cell regulation is mainly attributed to its role as a transcriptional regulator, which is achieved by direct binding of Oct4 to its canonical octamer motif through its DNA-binding domains 42,43. Interestingly, Oct4 contains two distinct DNA-binding domains and it can form either a homodimer with itself, or a heterodimer with other transcription factors, depending on the octamer half-sites present in the enhancer region of the target gene. Thus, it is interesting to investigate whether phosphorylation of Oct4 potentially affects its ability to form homodimers or heterodimers. To this end, we did not observe a significant effect of Akt-mediated Oct4 phosphorylation on Oct4 homodimer formation (Fig.1D). As generally Sox proteins require partners such as other transcription factors for activation and previous studies has demonstrated that Oct4 could form a complex with Sox2 on DNA to control the expression of embryonic development-related genes 44–47, next we examined whether Oct4 phosphorylation on T235 affects its interaction with Sox2. Interestingly, phosphorylation of Oct4 significantly reduced Oct4 interaction with Sox2 (Fig.1D). Consistent with this result, ectopic expression of Akt1 led to dissociation of Sox2 from wild-type Oct4 but not T235A Oct4 (Fig.1E). These data suggest that Akt-mediated phosphorylation of Oct4 on T235 might regulate cellular stemness in a signal dependent manner through modulating heterodimer formation with Sox2. Notably, a recent study illustrated that phosphorylation of Oct4 on T235 led to enhanced binding of Oct4 to Sox2 to differentially regulate transcription of stemness genes 39. The discrepancy between their observation and our study might stem from different cell lines examined, which might suggest that Akt-mediated phosphorylation of Oct4 at T235 might regulate transcription of stemness genes through modulating Oct4/Sox2 complex formation in a cellular context-dependent manner, and warrants further investigation. In addition, phosphorylation of Oct4 at S229 and Y327 has also been observed to have differential effects on its transcriptional activity toward multiple targets 48. Furthermore, Sox2 phosphorylation at T118 by Akt has also been reported to enhance the transcriptional ability of Sox2 in ESCs by unknown mechanisms 49. Similarly, phosphorylation of Nanog was observed in cell as well but has not been connected with any characterized function 50.

Furthermore, we observed that in addition to Oct4, Klf4 phosphorylation was also increased when co-overexpressed with Myr-Akt1 in cells (Fig.1F). By truncating Klf4 we narrowed down the Akt-mediated phosphorylation site(s) within amino acids 340–483, which contain an evolutionarily conserved AGC consensus motif located at T429. More importantly, mutation of Thr429 to Ala completely abolished the phosphorylation of the C-terminal portion of Klf4 by Akt (Fig.1F and G). As Klf4 could directly interact with the Oct4/Sox2 complex to facilitate somatic cell reprogramming 51, it is plausible that phosphorylation of Klf4 at T429, which resides in its zinc finger motif, might participate in modulating cellular stemness by affecting its interaction with the Oc4/Sox2 complex. Interesting, recent work has clearly demonstrated the critical role for Oct4 in pluripotency regulation, while Klf4 could be substituted by other factors 8,52, therefore we hypothesize that Akt controls the induced pluripotency process in large part by phosphorylation of Oct4, or by shifting Oct4-binding partners. Taken together, these results indicate that Akt-mediated phosphorylation might be an upstream regulatory mechanism responsible for the formation of iPS cells.

### Sox2, Oct4, and Klf4 are acetylated by p300 in vivo

**Figure 2:**
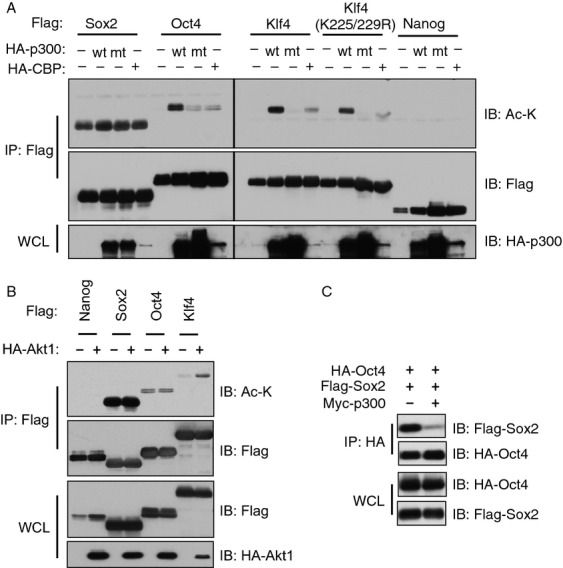
Oct4 and Klf4 are acetylated by p300 in vivo. (A) Whole cell lysates (WCLs) of 293T cells transfected with HA-tagged p300 or CBP with indicated Flag-tagged constructs were subjected to immunoprecipitation (IP) with Flag antibody. The Flag-IPs and WCLs were immunoblotted with indicated antibodies. Acetylation was detected by a Lys-acetylation (Ac-K) antibody. (B) WCLs of 293T cells transfected with indicated constructs were subjected to immunoprecipitation with Flag antibody. The Flag-IPs and WCLs were immunoblotted with indicated antibodies. (C) WCLs of 293T cells transfected with indicated constructs were subjected to immunoprecipitation with HA antibody. The HA-IPs and WCLs were immunoblotted with Flag and HA antibodies.

In addition to phosphorylation, various posttranslational modifications have been demonstrated as regulatory mechanisms in controlling the transcriptional activities of Oct4, Sox2, Klf4, and Nanog. For example, SUMOylation of Oct4 or Sox2 has been observed but with opposite effects on protein function. Specifically, Oct4 SUMOylation led to enhanced stability and DNA-binding ability 53, while Sox2 SUMOylation resulted in attenuated DNA-binding ability 54. As Akt has been demonstrated to modulate protein acetylation process by direct phosphorylation of the acetyl-transferases p300 55 or CBP 56, next we examined whether any of the four iPS factors, Oct4, Sox2, Klf4, or Nanog, were subjected to acetylation-mediated regulation. From our initial screening by coexpression of an iPS factor with either p300 or CBP in cells, we observed various acetylation patterns among these iPS factors. First, p300- or CBP-dependent acetylation of Nanog was not detected in our experimental condition by an Ac-K antibody (Fig.2A). Second, Sox2 displayed a high level of basal acetylation and a slight increase in acetylation in the presence of ectopic p300 or CBP (Fig.2A). Third, acetylation of both Oct4 and Klf4 was induced by p300-WT but not with a p300-acetyltransferase dead mutant, or CBP (Fig.2A). Interestingly, ectopic expression of Akt1 induced the acetylation of Klf4, but not Sox2, Oct4, or Nanog (Fig.2B), suggesting that the Akt activity directly and/or indirectly regulates acetylation state of iPS factors in a different mechanism. More importantly, p300-mediated acetylation of Oct4/Sox2 led to a dramatically decreased interaction between Sox2 and Oct4 (Fig.2C), suggesting that acetylation of Oct4/Sox2 behaves similarly to Oct4 phosphorylation-mediated impairment of the association between Oct4 and Sox2 (Fig.1D), which subsequently shifts the transcription activity of Oct4 toward a certain subset of genes 46,47.

### Sox2 is acetylated by p300 on multiple sites in vivo

**Figure 3:**
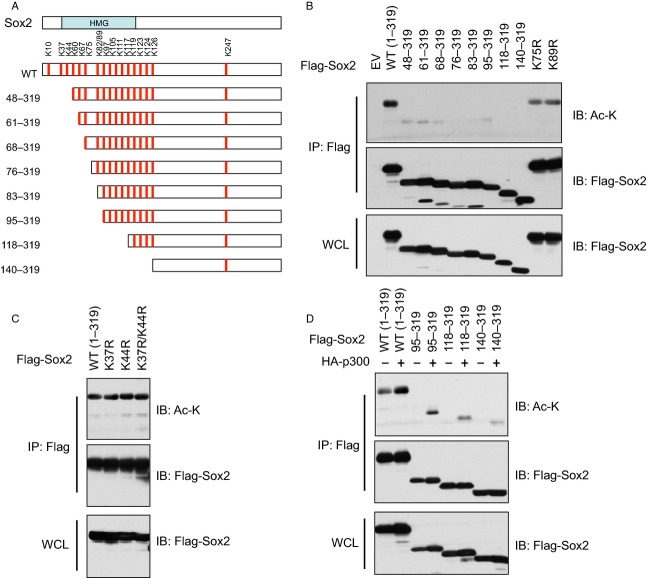
Mapping of Sox2 acetylation sites in vivo. (A) Schematic illustration of a series of generated Sox2 truncation mutations. (B) WCLs of 293T cells transfected with various Sox2 mutants were subjected to immunoprecipitation with Flag antibody. The Flag-IPs and WCLs were immunoblotted with Ac-K and Flag antibodies. (C) WCLs of 293T cells transfected with various Sox2 K-to-R substitution mutants were subjected to immunoprecipitation with Flag antibody. The Flag-IPs and WCLs were immunoblotted with Ac-K and Flag antibodies. (D) WCLs of 293T cells transfected with HA-p300 and various Sox2 truncation mutants were subjected to immunoprecipitation with Flag antibody. The Flag-IPs and WCLs were immunoblotted with Ac-K and Flag antibodies. WCLs, whole cell lysates; IP, immunoprecipitation.

To obtain mechanistic insights into how acetylation of Sox2 and Oct4 modulate their complex formation, we tried to pinpoint the acetylation sites on both Sox2 and Oct4. As Sox2 displayed both basal and p300-dependent acetylation events (Fig.2A), we first truncated Sox2 to narrow down the possible acetylation region(s) (Fig.3A). Interestingly, all truncations missing the first 1–48 amino acids showed dramatically reduced acetylation signals (Fig.3B), suggesting that the major basal Sox2 acetylation sites are located within the first 48 amino acids. As the HMG domain is the critical functional module for Sox2 function and there are two lysine residues (K37 and K44) located within this critical region, we further examined whether these two sites were the acetylation targets. Notably, mutation of K37, K44, or both sites to Arg to abolish possible acetylation did not significantly affect the basal acetylation state of Sox2 (Fig.3C), indicating that neither K37 nor K44 is the major acetylation site. In addition to K37 and K44, there is only one lysine, K10, left in the first 48 amino acids, thus it warrants further investigation to pinpoint whether K10 is the major site for Sox2 basal acetylation. Furthermore, for p300-dependent Sox2 acetylation, multiple sites might be involved, as a series of truncations displayed a gradual decrease in Sox2 acetylation (Fig.3D). One of these acetylation events on K75 has been recently reported to be critical to export Sox2 to the cytoplasm to terminate its transcriptional activity in the nucleus 57, which is consistent with our model that p300-mediated acetylation on Sox2/Oct4 impairs their ability to transcribe downstream genes.

### Oct4 is acetylated by p300 on multiple sites in cells

**Figure 4:**
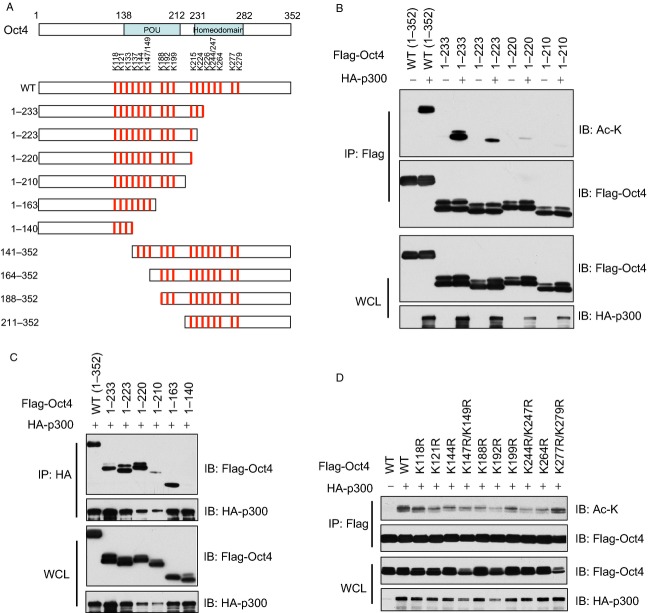
Mapping of Oct4 acetylation sites in vivo. (A) Schematic illustration of a series of generated Oct4 truncation mutations. (B and C) WCLs of 293T cells transfected with HA-p300 and various Oct4 truncation mutants were subjected to immunoprecipitation with Flag antibody. The Flag-IPs and WCLs were immunoblotted with indicated antibodies. (D) WCLs of 293T cells transfected with HA-p300 and various Oct4 K-to-R substitution mutants were subjected to immunoprecipitation with Flag antibody. The Flag-IPs and WCLs were immunoblotted with indicated antibodies. WCLs, whole cell lysates; IP, immunoprecipitation.

Similarly, we constructed a series of Oct4 truncation mutants to further map the acetylation sites on Oct4 (Fig.4A). In cells we observed that the acetylation sites were mainly located within amino acids 220–233 (Fig.4B), while the major p300 interacting motif was mapped within amino acids 140–163 (Fig.4C). As the POU and Homeodomain serve as a bipartite DNA-binding domain in Sox2 (Fig.4A), and part of the POU and HM domains are located within these two regions, we next generated single or double K-R mutations to examine which lysine was critical for Oct4 acetylation. Notably, all single KR mutation tested exhibited attenuated Oct4 acetylation status (Fig.4D), indicating that p300-mediated Oct4 acetylation occurs on multiple sites 16,58, which has been reported in many of p300 downstream substrates. Nevertheless, Oct4 and Sox2 acetylation led to impaired Oct4/Sox2 heterodimer formation (Fig.2C) and subsequent attenuated transcriptional activity 46,47.

### Klf4 is acetylated by p300 on multiple sites in cells

**Figure 5:**
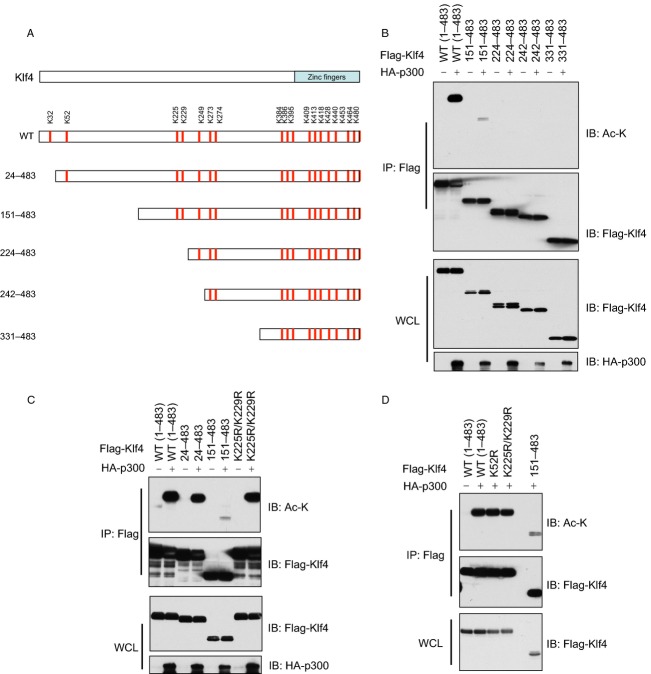
Mapping of Klf4 acetylation sites in vivo. (A) Schematic illustration of a series of Klf4 truncation mutations generated. (B) WCLs of 293T cells transfected with HA-p300 and various Klf4 truncation mutants were subjected to immunoprecipitation with Flag antibody. The Flag-IPs and WCLs were immunoblotted with indicated antibodies. (C and D) WCLs of 293T cells transfected with HA-p300 and various Klf4 truncation or K-to-R substitution mutants were subjected to immunoprecipitation with Flag antibody. The Flag-IPs and WCLs were immunoblotted with indicated antibodies. WCLs, whole cell lysates; IP, immunoprecipitation.

Furthermore, we generated Klf4 truncation mutations to pinpoint its p300-dependent acetylation site(s) (Fig.5A). By this method, we narrowed down the Klf4 acetylation sites to amino acids 1–151 (Fig.5B) and further to 25–151 (Fig.5C). Notably, a previous report indicated that p300 mainly acetylated Klf4 on K225 and K229 to enhance its transcriptional activity 59. However, in our experimental system, mutation of both K225 and K229 to Arg did not significantly reduce Klf4 acetylation status (Fig.5C), suggesting that there might be other sites involved in p300-mediated acetylation of Klf4. Consistent with this notion, we identified two lysine residues within amino acids 24–151, K32 and K52, that were possible acetylation sites (Fig.5D). However, mutating K52 to arginine did not noticeably affect Klf4 acetylation, suggesting that K32 or both K32 and K52 are possible p300 acetylation targets (Fig.5D) that warrant further investigation.

### Fbw7 possibly governs Klf4 stability in a GSK3-dependent manner

**Figure 6:**
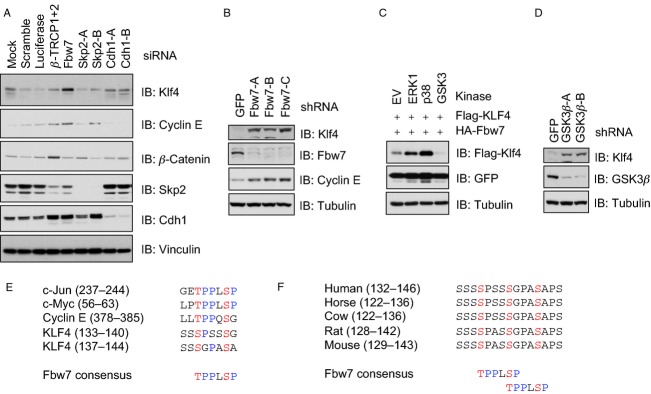
Fbw7 possibly governs Klf4 stability in a GSK3-dependent manner. (A) Fbw7-siRNA treatment in HeLa cells led to increased Klf4 expression. (B) Fbw7-shRNA treatments in HeLa cells led to Klf4 accumulation. (C) Overexpression of Fbw7 and GSK3 led to the destruction of Klf4. 293T cells were cotransfected with HA-Fbw7, Flag-Klf4 and indicated kinases and Klf4 abundance was measured by immunoblots with anti-Flag antibody. GFP was included as an internal transfection control and tubulin served as a loading control. (D) GSK3*β*-shRNA treatments in HeLa cells led to elevated level of Klf4. (E and F) Sequence alignment of the putative Fbw7 degrons in Klf4 (E) among different species (F).

During our examination of Klf4 acetylation, consistent with a previous report that Klf4 was subjected to 26S proteasome-mediated degradation 60, we observed that Klf4 was unstable. However, its upstream E3 ligases remain largely unknown. To this end, we screened a panel of E3 ligases for their possible roles in governing Klf4 stability by various siRNAs (Fig.6A). Interestingly, compared to the mock treatment, only depletion of Fbw7, but not other E3s examined, led to an accumulation of Klf4 (Fig.6A). The regulation of Klf4 by Fbw7 is further confirmed by the Fbw7 knockdown experiment with multiple independent shRNAs against Fbw7 (Fig.6B). As it is well characterized that Fbw7 only recognizes substrates with proper posttranslational modifications 61,62, next we attempted to identify its possible upstream modifying kinase(s). To this end, we observed that coexpression with GSK3, but neither ERK1 nor p38-MAPK, resulted in an efficient degradation of Klf4 (Fig.6C), and depletion of GSK3*β* led to accumulation of Klf4 (Fig.6D), demonstrating that GSK3 is a major upstream kinase responsible for Klf4 turnover mediated by Fbw7. This is consistent with a previous report that activation of the Akt pathway by peroxisome proliferator-activated receptor gamma agonist could stabilize Klf4 by reducing its ubiquitination 63. As phosphorylation of GSK3 by Akt can inactivate its kinase activity 64, which could lead to reduced Klf4 phosphorylation by GSK3, therefore evading Fbw7-mediated proteolysis. Through a close examination of the Klf4 protein sequence, we identified two putative Fbw7 consensus degrons 61 on Klf4 (Fig.6E) that are evolutionarily conserved (Fig.6F), which further supports Klf4 as a possible Fbw7 substrate and warrants further investigations.

## Discussion

Recent scientific advances have demonstrated that tumors arise in a step-wise fashion through gain-of-function mechanisms from certain oncogenes, concomitantly with the loss of expression of key tumor suppressor proteins 65,66. In addition to the p53 tumor suppressor pathway that is inactivated in about 50% of all human cancers, hyperactivation of the PTEN/PI3K/Akt pathway was observed in over 40% of human carcinomas 67,68. Recent work indicates that PTEN is directly linked to the hematopoietic stem cell renewal process 69,70. Furthermore, it is well established that mouse stem cells are committed to differentiation after withdrawal of the LIF ligand, indicating that a yet unknown downstream signal transduction pathway triggered by LIF is critical to maintain the stem cell state. Surprisingly, constitutive activation of Akt was shown to adequately substitute the function of LIF 71. However, it remains unclear how constitutive activation of Akt signaling is sufficient to maintain pluripotency.

**Figure 7:**
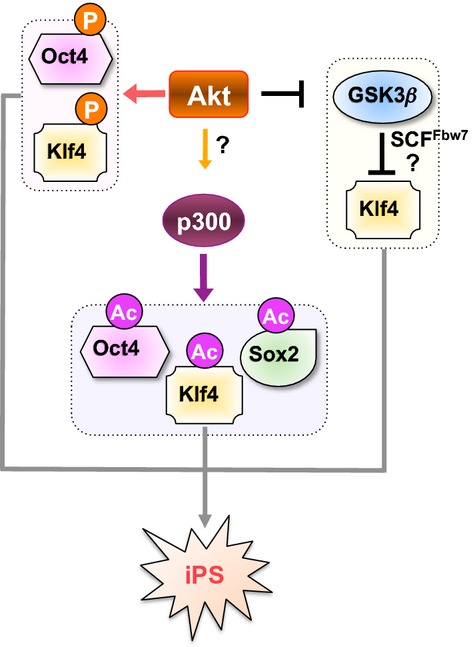
A schematic model for how Akt mediates the induced pluripotent stem (iPS) process through direct or indirect regulation of posttranslational modifications of iPS-inducing factors. Akt directly phosphorylates Oct4 to modulate its interaction with Sox2, leading to a shift of Oct4/Sox2-mediated transcription events. Akt may also activate p300 to promote acetylation of Oct4, Sox2, and Klf4 at multiple sites to change their transcription activity. Furthermore, Akt phosphorylates GSK3, resulting in reduced Klf4 phosphorylation by GSK3 that could possibly trigger Fbw7-mediated degradation of Klf4.

Oct4, Sox2, and Klf4 co-occupy a substantial portion of their downstream target genes to build up a regulatory circuit consisting of multiple autoregulatory and feed-forward loops. In this scenario, it creates consistent activity above a threshold level to maintain the pluripotent state, while the whole system is unstable, and might quickly shut down when one critical regulator becomes negative 72,73. To this end, we observed that Akt directly phosphorylated key transcriptional factors including Oct4 and Klf4 to restrict their activities below this threshold. These results also indicate that Akt-mediated phosphorylation might be an upstream regulatory mechanism responsible for the formation of iPS cells (Fig.7). Therefore, it will be intriguing to examine whether coexpression of Myr-Akt (an active form of Akt) together with Sox2, Oct4, and Klf4 will greatly enhance the efficiency of iPS formation. Furthermore, whether point mutations of all the potential Akt sites in Oct4 or Klf4 would attenuate this phenotype.

In addition to a direct role of Akt-mediated phosphorylation of iPS factors in regulating the iPS process, Akt could also indirectly affect stemness by modulating other posttranslational modifications of iPS factors. To this end, we have identified that Akt could either facilitate p300-mediated acetylation of Oct4, Sox2, and Klf4 by directly activating p300, or stabilizing Klf4 by directly phosphorylating and inactivating GSK3 to evade Fbw7-mediated degradation of Klf4 (Fig.7). Consistently, multilayer regulations of iPS factors have been identified to cooperatively regulate the pluripotency. For example, a chemical agent ATRA has been shown to increase the interaction of Klf4 with p300 by inducing Klf4 phosphorylation via activation of c-Jun N-terminal kinase and p38 MAPK signaling, and Klf4 acetylation by p300 increased its activity to transactivate the Mfn-2 promoter 74. Since Oct4, Sox2, and Klf4 were found to be key regulators of stem cells, it is possible that Akt could directly or indirectly modulate their transcriptional activities, thus influencing the maintenance of pluripotency.

As cellular reprogramming and the carcinogenic process share many similar features and mechanisms 75,76, it is not surprising that these iPS markers might play critical roles in tumorigenesis as well. To this end, overexpression of Sox2 77–79 or Oct4 80–82 has been observed in multiple cancer types. Similarly, Klf4 overexpression was observed to promote malignant transformation through downregulation of the Cdk inhibitor p21 83. Klf4 belongs to the family of Kruppel-like transcription factors, whose functions have been implicated in regulation of tissue-specific development 84. Elevated Klf4 overexpression is frequently observed in many types of cancers 85 and overexpressing Klf4 in mice led to squamous cell cancer 86, while the molecular mechanisms remain unclear. However, on the other hand, recent studies demonstrated that rather than an oncogene, Klf4 serves as an inhibitor for tumor cell growth and migration 85,87,88. Interestingly, loss of *Fbw7* is frequently found in T-cell acute leukemia (T-ALL), a disease caused by the blockage of proper differentiation from progenitor cells to mature T cells. In this study, we found that Fbw7 could possibly degrade Klf4 in a GSK3-dependent manner. As a result, loss of Fbw7 could cause accumulation of the Klf4 transcription factor, which might subsequently block the proper differentiation process, leading to the development of leukemia. Taken together, our study provided insight into the critical role of the Akt oncogenic pathway in regulating stem cell reprogramming and impact on the cancer stem cells. Thus, it will provide the rationale, therefore opening new avenues for developing Akt-specific inhibitors as efficient anticancer drugs.
